# Reframing the one health approach to antimicrobial resistance: The epidemiologic significance of environmental water quality

**DOI:** 10.1016/j.jtumed.2026.01.001

**Published:** 2026-01-18

**Authors:** Christian Joseph N. Ong, Jerico B. Ogaya, Rahmatullah Nazari, Mohamed M. Ahmed, Don E. Lucero-Prisno

**Affiliations:** a Department of Biology, College of Science, De La Salle University, Manila, Philippines; b Center for University Research, University of Makati, Makati City, Philippines; c Research Services Office, Palompon Institute of Technology, Leyte, Philippines; d Department of Medical Technology, Institute of Health Sciences and Nursing, Far Eastern University, Manila, Philippines; e Faculty of Dentistry, Cheragh University of Medical Sciences, Kabul, Afghanistan; f Faculty of Medicine and Health Sciences, SIMAD University, Mogadishu, Somalia; g Department of Global Health and Development, London School of Hygiene and Tropical Medicine, London, United Kingdom

Dear Editor,

The increasing prevalence of antimicrobial resistance (AMR) is a serious, self-inflicted public health crisis. Although the ‘One Health’ framework highlights the crucial connections between human, animal, and environmental health, our current surveillance and intervention strategies remain heavily focused on clinical practices. As clinical epidemiologists and global health professionals, we must urgently shift our perspective to address the often-overlooked role of the environment, particularly water quality, in perpetuating the AMR problem. Currently, AMR surveillance largely concentrates on tracking resistance trends within advanced healthcare institutions and patient populations.^1^ While this focus is vital for guiding short-term clinical practices, it overlooks a significant epidemiological gap: the ongoing, unmeasured spread of community-acquired multidrug-resistant infections (MDRIs). Central to epidemiological investigations is the need to pinpoint the primary sources and key transmission dynamics of resistance, which frequently implicate contaminated water sources.

This issue is particularly exacerbated in low- and middle-income countries (L&MICs), where untreated domestic, industrial, and agricultural wastewater is often released into the environment.^2^ This leads to surface water bodies becoming densely populated with antibiotic-resistant bacteria (ARB) and antibiotic resistance genes (ARGs) originating from human and animal waste. Consequently, this polluted water is often used for critical daily activities, such as drinking, bathing, and irrigating crops, effectively transforming these water sources into significant conduits for resistance transmission.^3^ Such environmental contamination represents an ongoing, indiscriminate exposure to resistance that undermines local efforts in antibiotic stewardship and infection control.^4^ For clinical epidemiologists, this highlights a crucial insight: the level of resistance in the environment shapes the baseline of community resistance, making patient-focused interventions much less effective. How can we expect to see real progress in reducing community-acquired resistance when the public is continuously exposed to high concentrations of resistance genes through routine environmental interactions? Moreover, outbreaks of drug-resistant pathogens, such as Vibrio cholerae or Salmonella typhi, become increasingly challenging and costly to manage, placing immense pressure on already strained healthcare systems.^5^

To align with the journal's mission of enhancing health practices through evidence, we must advocate for an urgent epidemiological shift in the field. The conventional model of monitoring health outcomes should be broadened to include a rigorous assessment of environmental factors. This requires the essential integration of water resistome surveillance—measuring ARB and ARG levels in significant surface and groundwater sources—into national and regional clinical epidemiology frameworks.^6^^,^^7^ This information is crucial for identifying high-risk areas for transmission and implementing targeted interventions. For global health policy, the most effective “clinical” strategy against AMR in L&MICs might not be the introduction of new medications or prescribing guidelines, but rather a comprehensive global investment in resilient water, sanitation, and hygiene (WASH) infrastructure.^8^ Analyzing the costs and benefits in terms of lives saved and healthcare costs avoided from managing MDRIs strongly favors this infrastructural approach over merely focusing on pharmaceutical solutions.^9^

Our battle against AMR is fundamentally limited by the chosen measurement and intervention strategies. By recognizing and incorporating the clinical epidemiological implications of environmental water quality, we can open a crucial and lasting path toward global health security. Now is the time to embrace a holistic, evidence-based One Health approach that places environmental infrastructure at its core.

## Consent to publish

Not applicable.

## Consent to participate

Not applicable.

## Ethical approval

Not required for this study.

## Authors contributions

CJNO conceptualized and designed the study; JBO and RN performed the literature review and data curation; CJNO and MMA wrote the manuscript; DELP III revised the manuscript; and all authors have read and approved the final manuscript. All authors have critically reviewed and approved the final draft and are responsible for the content and similarity index of the manuscript.

## Data availability

Not applicable; no new datasets were generated or analyzed.

## Source of funding

This research did not receive any specific grant from funding agencies in the public, commercial, or not-for-profit sectors.

## Conflict of interest

The authors have no conflict of interest to declare.
